# Dual-Emissive Waterborne Polyurethanes Prepared from Naphthalimide Derivative

**DOI:** 10.3390/polym9090411

**Published:** 2017-09-03

**Authors:** Tao Wang, Xingyuan Zhang, Yipeng Deng, Wei Sun, Qidong Wang, Fei Xu, Xiaowen Huang

**Affiliations:** CAS Key Laboratory of Soft Matter Chemistry, Department of Polymer Science and Engineering, University of Science and Technology of China, Hefei 230026, China; wt0510@mail.ustc.edu.cn (T.W.); dengyp@mail.ustc.edu.cn (Y.D.); kdsw@mail.ustc.edu.cn (W.S.); wqd1990@mail.ustc.edu.cn (Q.W.); puglobe@mail.ustc.edu.cn (F.X.); huangxw@mail.ustc.edu.cn (X.H.)

**Keywords:** fluorescence, room-temperature phosphorescence, dual-emissive, waterborne polyurethane, halogen atom effect, excimers

## Abstract

Fluorescent and room-temperature phosphorescent (RTP) materials are widely used in bioimaging, chemical sensing, optoelectronics and encryption. Here, a series of single-component dual-emissive waterborne polyurethanes (WPUs) with both fluorescence and room-temperature phosphorescence were synthesized. Dye without halogen atom incorporated into WPUs can only exhibit fluorescence due to poor spin-orbit coupling. When bromine atom is introduced into dye, we found that WPUs can emit both fluorescence and room-temperature phosphorescence with lifetimes up to milliseconds because of enhanced spin-orbit coupling. Moreover, with an increase in dye concentrations in WPUs, excimers are formed due to the aggregation effect, and may promote communication between singlet and triplet states. At different dye concentrations, structural, thermal, and luminescent properties serve as the main focus.

## 1. Introduction

Fluorescent and phosphorescent materials are extensively used in bioimaging [1,2,3], sensors [4,5,6] and organic light-emitting diodes (OLEDs) [7,8,9]. Generally, purely organic dual-emissive materials with both fluorescent and room-temperature phosphorescent (RTP) materials are rare, because thermal decay and oxygen can quench phosphorescence. In recent years, dual-emissive materials have been used as sensors for molecular oxygen by monitoring the ratio of intensity of fluorescence and room-temperature phosphorescence [10,11,12]. In electroluminescent devices, 25% singlet excitons and 75% triplet excitons are produced after the recombination of electrons and holes, according to spin statistics [13,14]. Therefore, RTP materials are significant for OLEDs. Moreover, RTP materials have a potential application in encryption ink [15]. Generally speaking, organometallic molecules may more easily exhibit RTP properties, because heavy metal ions enhance spin-orbit coupling or decrease the small energy gap between singlet and triple states caused by charge transfer of metal to ligand (MLCT) or ligand to metal (LMCT) [16]. However, organometallic materials face some difficulties in application, due to their toxicity and instability in aqueous conditions. Nowadays, many researchers are paying a great deal of attention to developing purely organic RTP materials that can, to a large extent, avoid the problems brought by organometallic systems.

In order to decrease thermal decay and quenching by oxygen, luminescent dyes are made as crystals, or are blended into polymers [11,17,18,19]. For example, Kim et al. [20] prepared a series of organic crystals that could emit bright phosphorescence at room temperature due to halogen bonding. However, it is difficult to maintain a crystalline structure in real applications. Another strategy for blending dyes into a rigid matrix could cause phase separation when dye concentration reaches a certain level. Fraser et al. [21,22] synthesized plenty of dye-initiated dual-emissive polymers, which are able to effectively avoid phase separation. Nevertheless, end-functionalized dyes are prone to aggregate, and are more susceptible to thermal quenching because of the increased free volumes. Fortunately, the fatal problem can be avoided when the dyes are incorporated into the main chain, due to the limitation of molecular motions [23]. Therefore, waterborne polyurethanes (WPUs) prepared by polycondensation reactions may be a desirable alternative for generating polymers while, at the same time, not decreasing the molecular weight when the percentage of dyes increases compared to end-functionalized polymers. Recently, waterborne polyurethanes (WPUs) have been used in leathers, foams, paintings, textiles, coatings, thermoplastic elastomers [24,25,26] because of the feasible adjustment of soft segments and hard segments according to practical needs.

Herein, we report a series of single-component dual-emissive WPUs from a naphthalimide derivative with both fluorescence and room-temperature phosphorescence by a facile reaction. In this article, we will focus on the synthesis and optical characterization of WPUs.

## 2. Experimental

### 2.1. Materials

1,8-Naphthalenedicarboxylicanhydride, 4-bromo-1,8-naphthalene anhydride and 2-Amino-1,3-propanediol were purchased from Energy Chemical Reagent Co., Ltd. (Shanghai, China). Isophorone Diisocyanate (IPDI), polytetramethylene glycol (PTMG, *M*_n_ = 2000), 1,4-butanediol (BDO), 2,2-dimethylol propionic acid (DMPA), Dibutyltin dilaurate (DBTDL) and trimethylamine were obtained from Aladdin (Shanghai, China). Other reagents were received from Sinopharm Chemical Reagent Co., Ltd. (Shanghai, China) and used as received.

### 2.2. Methods

^1^H and ^13^C NMR were recorded on a Bruker AV300 and Bruker AV400 NMR spectrometer (Bruker Co., Ltd., Karlsruhe, Germany) operated in the Fourier transform mode using TMS as internal standard in *d*_6_-DMSO. FTIR spectra were recorded on a Bruker Tensor27 FTIR spectrometer (Bruker Co., Ltd., Karlsruhe, Germany) in the range of 4000–400 cm^−1^. UV-VIS-NIR absorption spectra were obtained from a SOLID3700 UV-VIS-NIR spectrometer (Shimadzu Co., Ltd., Kyoto, Japan) ranging from 240–1200 nm. Differential scanning calorimetry (DSC) measurements were conducted at 10 °C/min under nitrogen atmosphere on Mettler-Toledo DSC (Mettler-Toledo Co., Ltd., Zurich, Switzerland). Thermogravimetric (TG) analysis spectra were investigated on Shimadzu TGA-50 (Shimadzu Co., Ltd., Kyoto, Japan) at 10 °C/min under nitrogen atmosphere in the range of 10–700 °C. Gel permeation chromatography (GPC) measurements were recorded on HP1100 (Waters Co., Ltd., Milford, Massachusetts, America) using THF as eluent with a flow rate of 0.3 mL/min^−1^, calibrated with linear polystyrene standards. MALDI-TOF mass spectra (ESI) were recorded on the Atouflex Speed mass spectrometer (Bruker Co., Ltd., Karlsruhe, Germany). Steady-state fluorescence emission spectra were measured with a FluoroMax-4 spectrafluorometer (Horiba Jobin Yvon Co., Ltd., Paris, France) and analyzed with an Origin integrated software FluoroEssence (v2.2) (Horiba Jobin Yvon Co., Ltd., Paris, France). Fluorescent and phosphorescent lifetime data were obtained with a 1MHz LED laser with the excitation peak at 374 nm (NanoLED-370 and SpectraLED-370) (Horiba Jobin Yvon Co., Ltd., Paris, France) and analyzed with DataStation v6.6 (Horiba Jobin Yvon Co., Ltd., Paris, France).

### 2.3 Synthesis

Preparation of 2-(1,3-dihydroxypropan-2-yl)-1H-benzo[de]isoquinoline-1,3(2H)-dione (NI): 1,8-naphthalic anhydride (5 g, 25.3 mmol), 2-Amino-1,3-propanediol (2.73 g, 30.0 mmol) and 250 mL ethanol were added to a round-bottom flask equipped with a magnetic stir bar and a condenser. Then the reaction was conducted under reflux overnight. After the reaction finished, the mixture was then cooled and filtered. Then the solid was collected. The solid was further crystallized by ethanol and the desired compound was obtained as a white crystal (5.47 g, 80%). ^1^H NMR (400 MHz, DMSO-*d*_6_), δ (TMS, ppm): 8.47 (ddd, *J* = 13.5, 7.8, 1.2 Hz, 4H), 7.87 (dd, *J* = 8.2, 7.3 Hz, 2H), 5.24 (tt, *J* = 8.0, 5.9 Hz, 1H), 4.80 (dd, *J* = 6.6, 5.4 Hz, 2H), 4.00–3.78 (m, 4H). ^13^C NMR (100MHz, DMSO-*d*_6_), δ (TMS, ppm): 164.73, 134.35, 131.62, 131.01, 127.65, 123.1, 59.43, 58.54. MS (ESI): *m*/*z* [M + Na]^+^ Calcd for C_15_H_14_O_4_NNa 294.07368, found 294.07440 (Figures S1–S3).

Preparation of 6-bromo-2-(1,3-dihydroxypropan-2-yl)-1H-benzo[de]isoquinoline-1,3(2H)-dione (NIBr)**:** The same method as NI with 4-Bromo-1,8-naphthalic anhydride instead of 1,8-naphthalene anhydride. 5.37g, 85%. ^1^H NMR (300 MHz, DMSO-*d*_6_), δ (TMS, ppm): 8.66–8.49 (m, 2H), 8.34 (d, *J* = 7.9 Hz, 1H), 8.24 (d, *J* = 7.9 Hz, 1H), 8.01 (dd, *J* = 8.5, 7.3 Hz, 1H), 5.29–5.13 (m, 1H), 4.80 (dd, *J* = 6.7, 5.3 Hz, 2H), 3.96 (ddd, *J* = 11.1, 8.0, 5.3 Hz, 2H), 3.80 (dt, *J* = 11.1, 6.3 Hz, 2H). ^13^C NMR (75 MHz, DMSO-*d*_6_), δ (TMS, ppm): 163.62, 132.15, 131.40, 131.24, 130.81, 129.55, 128.71, 128.61, 128.38, 123.28, 122.50, 58.85, 58.26. MS (ESI): *m*/*z* [M + H]^+^ Calcd for C_15_H_13_O_4_NBr 350.00225, found 350.00223 (Figures S4–S6).

Preparation of waterborne polyurethanes (WPUs): PTMG was added to a 100 mL three-neck round-bottom flask equipped with a mechanical stirrer, thermometer and condenser. The reaction was first conducted at 100 °C to eliminate water under condition of reduced pressure for twenty minutes. Then IPDI was added to the flask under 90 °C and allowed to react for one hour. Subsequently, DMPA was added and heated at 80 °C for one hour and then NI (or NIBr) and BDO were added at 80 °C for two hours. After a drop of DBTDL was added, the reaction was conducted at 70 °C for five hours. In the course of polymerization, a moderate amount of acetone was added to reduce the viscosity. When the polymerization finished, Triethylamine (TEA) as a neutralization agent was added under 40 °C reacting with carboxyl of DA to form quaternized NCO-terminated polyurethane. Finally, water was poured into the flask with high shearing speed to emulsify the polyurethane for 30 min. 

## 3. Results and Discussion

**Scheme 1:**
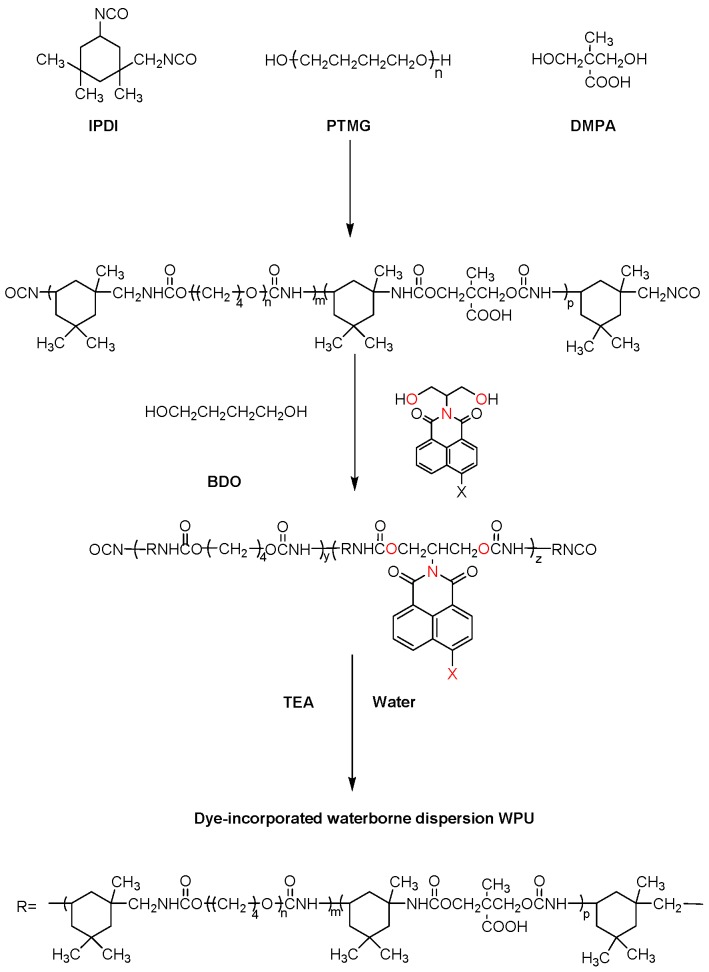
Synthetic process of dye-incorporated WPU.

The synthetic process of WPU is shown in Scheme 1. As is well known, metal-free luminescent molecules barely generate room-temperature phosphorescence. Besides thermal decay and quenching by oxygen, another significant reason is inefficient spin-orbit coupling (SOC). In order to solve these thorny problems, NI and NIBr were incorporated into WPUs to decrease thermal decay and enhance SOC by halogen atom.

### 3.1. Structural Characterization of WPUs by FTIR

**Figure 1:**
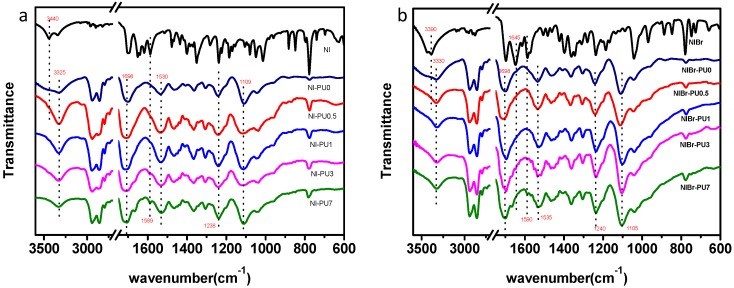
FTIR spectra of NI-PU (**a**) and NIBr-PU (**b**) films with different dye ratios (e.g., NI-PU1 means 1% NI by weight in WPU).

Figure 1 shows the FTIR of dye-incorporated WPUs. The characteristic absorption peak at 3390–3440 cm^−1^ ascribed to O–H stretching vibration disappears in the WPUs, which means that the NI and NIBr are incorporated covalently. With an increase in the concentrations of dyes, the characteristic peaks at 1589 cm^−1^ (for NI-PU), 1590 and 1645 cm^−1^ (for NIBr-PU) belonging to phenyl rings skeleton vibration appear, which further demonstrates that the dyes have been chemically linked to the WPUs. Other peak assignments for dye-incorporated WPUs: 3325 and 3330 cm^−1^ (ν_NH_), 2940–2860 cm^−1^ (ν_CH2_ and ν_CH3_), 1696 cm^−1^ (ν_C=O_), 1530 and 1535 cm^−1^(δ_N-H_), 1238 and 1240 cm^−1^ (ν_C–O_ in carbamate group), 1105 and 1109 cm^−1^ (ν_C–O–C_ in soft segment). In order to demonstrate that the dyes are incorporated into WPUs completely, detailed NMR analyses can be found in the supporting information (Figure S7).

### 3.2. Molecular Weight Measurements for WPUs

The molecular weight information for WPUs is characterized by gel permeation chromatography (GPC) (Table 1, Figures S12 and S13). The polydispersity indices are around 2; a typical feature of polycondesation reactions. It is noteworthy that number-average molecular weights of WPUs are not reliable because of interaction between extremely polar ammonium groups (or carboxylate groups) and the elution column.

### 3.3. DSC and TG Analyses

**Figure 2:**
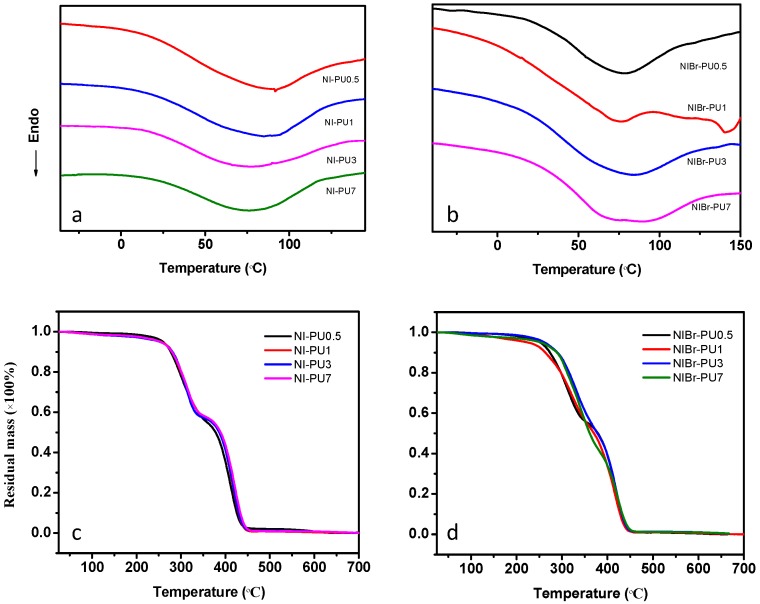
DSC curves for NI-PU (**a**) and NIBr-PU (**b**). TG analysis spectra of NI-PU (**c**) and NIBr-PU (**d**).

Shown in Figure 2 are the thermal properties of WPU films. As the DSC curves (Figure 2a,b) show, all of samples exhibit a distinct broad endothermal peak in the range 50 from 100 °C (not the typical glass transition), which might be caused by the disruption of the relative short-range order in hard segments [27]. It is worth noting that there is another endothermal peak for NIBr-PU1 in the range of 130–150 °C due to the melting of well-ordered microcrystalline structures formed in the course of film formation. According to thermogravimetry analysis (Figure 2c,d), there are three obvious stages in the course of WPUs decomposition. All of the samples show a slow descending slope from the onset to 250 °C, which is ascribed to the elimination of small molecules such as water and triethylamine. A sharp descent in the range of 250–345 °C is due to the breaking of carbamate and allophanate bonds. The last stage between 345 and 450 °C is the decomposition of C–C bonds in the soft segments. With the increase in dye content, thermostability should improve because of rigid dye molecules, which may be confirmed by the continually increasing maximum decomposition temperature (Figure S8).

### 3.4. Luminescent Properties

**Figure 3:**
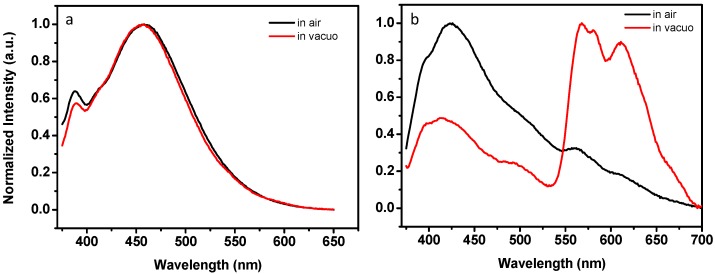
Steady-state emission spectra (298 k) of NI-PU3 (**a**) and NIBr-PU3 (**b**) in air (black line) and in vacuo (red line) (λ_ex_ = 365 nm).

**Figure 4:**
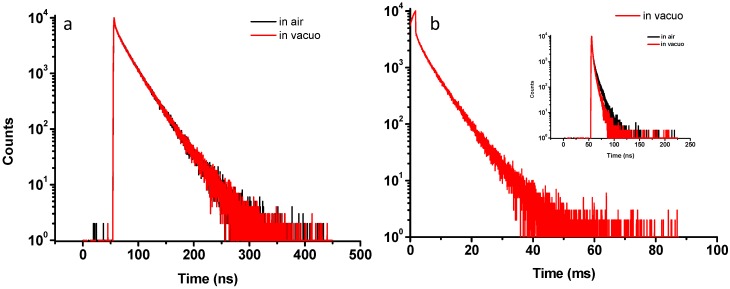
Photoluminescence lifetime decay (λ_ex_ = 374 nm) in air and in vacuo at 298 K: NI-PU3 ((**a**) λ_em_ = 456 in air and 457nm in vacuo) NIBr-PU3 (b, λ_em_ = 568 nm in vacuo). Inset of (**b**): photoluminescence lifetime decay of NI-PU3 (λ_em_ = 426 in air and 420 nm in vacuo).

The optical properties are mainly investigated in the solid film, because its applications are mostly in the solid state. As Figure 3a shows, the steady-state spectrum of NI-PU3 film in air at 298 K exhibits a band around 456 nm with a lifetime of 24.8 ns (Figure 4, Table S1), which is a typical characteristic of fluorescence. However, when the film is sealed in a vacuum tube, its steady-state spectrum (λ_em_ = 457 nm, τ = 24.7 ns) is similar to that under air, without obvious change. As a result, we can confirm that NI-PU3 only has fluorescence, and not the expected room-temperature phosphorescence. The reason why NI-PU3 cannot exhibit RTP properties may be inefficient spin-orbit coupling, which causes weak intersystem crossing. It is noteworthy that the peak around 456 nm may belong to excimers and the shoulder peak is the emission from original excitons. In order to induce room-temperature phosphorescence, we introduce bromine atom into NI (NIBr) to enhance spin-orbit coupling.

**Figure 5:**
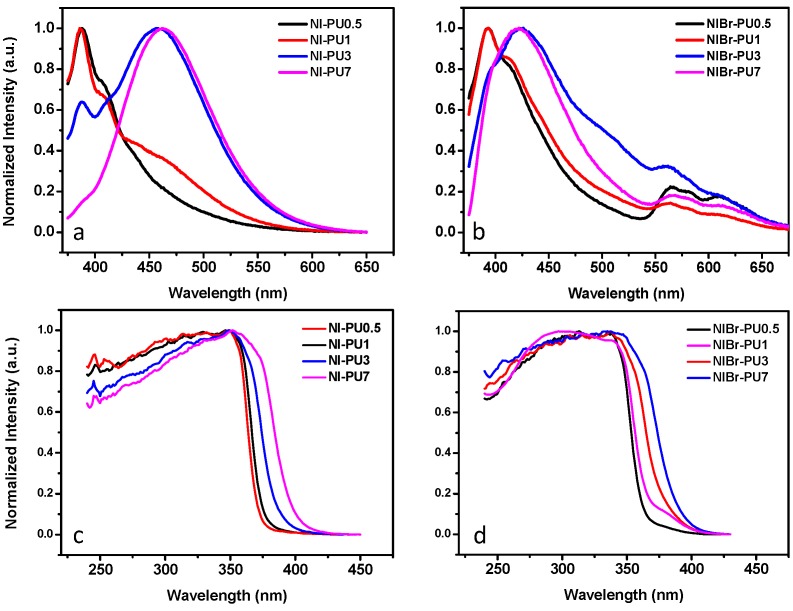
Steady-state emission spectra of different NI (**a**) and NIBr (**b**) concentrations in WPUs under air (λ_ex_ = 365 nm); Absorption spectra of different NI (**c**) and NIBr (**d**) concentrations in WPUs.

As shown in Figure 3b, NIBr-PU3 shows a maximum fluorescent emission peak centered at 426 nm (τ = 2.9 ns, Table S2) under air. When NIBr-PU3 is sealed in vacuo, apart from the band around 420 nm (τ = 2.1 ns), a new structured band appears around 530–700 nm. The structured peak is ascribed to the vibration progress of NIBr molecules. Moreover, the lifetime of the film in vacuo is 4.95 ms (λ_em_ = 568 nm). Therefore, it is confirmed that the band around 530–700 nm belongs to phosphorescence, whose lowest excited triplet state is a π-localized excited state ^3^(π–π)*. That is to say, NIBr-PU3 has both fluorescence and room-temperature phosphorescence. We also investigated different incorporated concentrations of dyes in WPUs. With increased dye loadings, besides a peak around 390 nm, a new band around 455 nm for NI-PU and 420 nm for NIBr-PU appears (Figure 5a,b; Figure S9). The steady-state emission spectra of NI-PU emulsions have the similar phenomenon (Detailed discussion is presented in the supporting information, Figure S10, Table S3). This may be caused by the formation of excimers, which are usually accompanied by bathochromic effect [28,29]. Therefore, excimers may promote communication between singlet and triplet states to some extent, on account of interactions in singlet-state excitons and ground-state molecules, which in general decrease the energy of singlet-state excitons.

The optical energy gap (Δ*E*_g_) from onset of absorption decreases as the concentrations of dyes increase, which may be ascribed to dye-dye interaction in ground state (Figure 5c,d). To the best of our knowledge, the formation of excimers is due to interaction between excitons and ground-state molecules. Therefore, excimers don’t evidently change the absorption spectra of luminophores. As for the absorption spectra of NI-PU and NIBr-PU, the maximum absorption peaks are almost similar to each other (the absorption spectra of NI-PU emulsions are also similar, Figure S11), which means dye-incorporated WPUs have the same excited pathway. As a result, the appearance of the new bands (455 nm for NI-PU, 420 nm for NIBr-PU) could be attributed to the formation of the excimers. Compared to the steady-state emission spectra for NIBr-PU in vacuum (Figure 5b, Figure S9), the emission spectra under air have weak emission peaks around 530–700 nm, which are ascribed to phosphorescence. This may be an enhanced triplet-to-ground-state decay rate due to the heavy atom effect of bromine, which outcompetes the relatively slow rate of oxygen diffusion in WPU films.

NIBr-PU may make possible a number of the enormous potential applications of single-component dual-emissive materials in oxygen sensors, OLEDs and encryption ink. In particular, polymer OLEDs have been a hot topic due to their facile processing controls and absence of aggregation-caused quenching in comparison to small-molecule OLEDs. In our next works, we will focus on application in polymer OLEDs.

## 4. Conclusions

In conclusion, a series of purely organic single-component dual-emissive materials with both fluorescence and room-temperature phosphorescence were prepared by incorporating naphthalimide derivative into waterborne polyurethanes. By comparing NI-PU with NIBr-PU, we have demonstrated that stable dual-emissive polymers can be easily accessible by the halogen atom effect without sophisticated synthesis. With increased dye contents, excimers would be formed due to interaction between singlet-state excitons and ground-state molecules, which may promote the communication between singlet and triplet states to generate room-temperature phosphorescence.

## Figures and Tables

**Table 1:** GPC Data of NI-PU and NIBr-PU films.

Sample ^a^	wt % ^b^	*M*_n_^c^	*M*_w_^d^	PDI ^e^
NI-PU0.5	0.5	1.8 × 10^4^	4.1 × 10^4^	2.3
NI-PU1	1	2.7 × 10^4^	5.2 × 10^4^	1.9
NI-PU3	3	1.8 × 10^4^	4.0 × 10^4^	2.2
NI-PU7	7	2.3 × 10^4^	4.1 × 10^4^	1.8
NIBr-PU0.5	0.5	2.1 × 10^4^	4.5 × 10^4^	2.1
NIBr-PU1	1	1.3 × 10^4^	2.4 × 10^4^	1.8
NIBr-PU3	3	1.7 × 10^4^	3.8 × 10^4^	2.2
NIBr-PU7	7	2.2 × 10^4^	5.6 × 10^4^	2.5

^a^ All samples were prepared at the PTMG:IPDI:DMPA ratio of 1:5:2.9; ^b^ Weight percentage of NI and NIBr; ^c^ Number-average molecular weight measured in THF; ^d^ weight-average molecular weight measured in THF; ^e^ Polydispersity index (*M*_w_/*M*_n_).

## Supplementary Materials

The following are available online at www.mdpi.com/2073-4360/9/9/411/s1. **Figure S1.** ^1^H NMR spectrum of NI in DMSO; **Figure S2.** ^13^C NMR spectrum of NI in DMSO; **Figure S3.** MALDI-TOF-MS of NI; **Figure S4.** ^1^H NMR spectrum of NIBr in DMSO; **Figure S5.** ^13^C NMR spectrum of NIBr in DMSO; **Figure S6.** MALDI-TOF-MS of NIBr; **Figure S7.** NMR analysis of NI-PUs (a) and NIBr-PUs in *d*_6_-DMSO; **Figure S8.** Derivative thermogravimetric (DTG) analysis of NI-PUs (a) and NIBr-PUs (b); **Figure S9.** Steady-state emission spectra of different NI (a) and NIBr (b) concentrations in WPU under vacuum; **Figure S10.** Normalized steady-state emission spectra of NI-PUs (λ_ex_ = 350 nm) in the emulsion state at room-temperature; **Figure S11.** UV-Vis absorption spectra of NI-PUs in the emulsion state; **Table S1.** lifetime data for NI-PUs in air and vacuum under room temperature; **Table S2.** Lifetime data for NIBr-PUs in air and vacuum under room temperature; **Table S3.** Luminescent data for NI-PU emulsions in air at room temperature; **Figure S12.** GPC traces for NI-PU0.5 (a), NI-PU1 (b), NI-PU3 (c), NI-PU7 (d) in THF; **Figure S13.** GPC traces for NIBr-PU0.5 (a), NIBr-PU1 (b), NIBr-PU3 (c), NIBr-PU7 (d) in THF with a flow rate of 0.3 mL/min^−1^, calibrated with linear polystyrene standards.
