# Supplementary material for: Dual-Emissive Waterborne Polyurethanes Prepared from Naphthalimide Derivative
Source: Polymers (Basel). 2017 Sep 3;9(9):411. doi: 10.3390/polym9090411 (PMC6418983; doi:10.3390/polym9090411)
Supplement: Supplementary file 1 [file polymers-09-00411-s001.pdf]

polymers

Electronic supplementary Information for Dual-Emissive Waterborne Polyurethanes Prepared from Naphthalimide Derivative

Tao Wang, Xingyuan Zhang*, Yipeng Deng, Wei Sun, Qidong Wang, Fei Xu, Xiaowen Huang

CAS Key Laboratory of Soft Matter Chemistry, Department of Polymer Science and Engineering, University of Science and Technology of China, Hefei, 230026, P.R.China.
* Corresponding author: zxym@ustc.edu.cn (Xingyuan Zhang)

Supplementary Figures and Tables

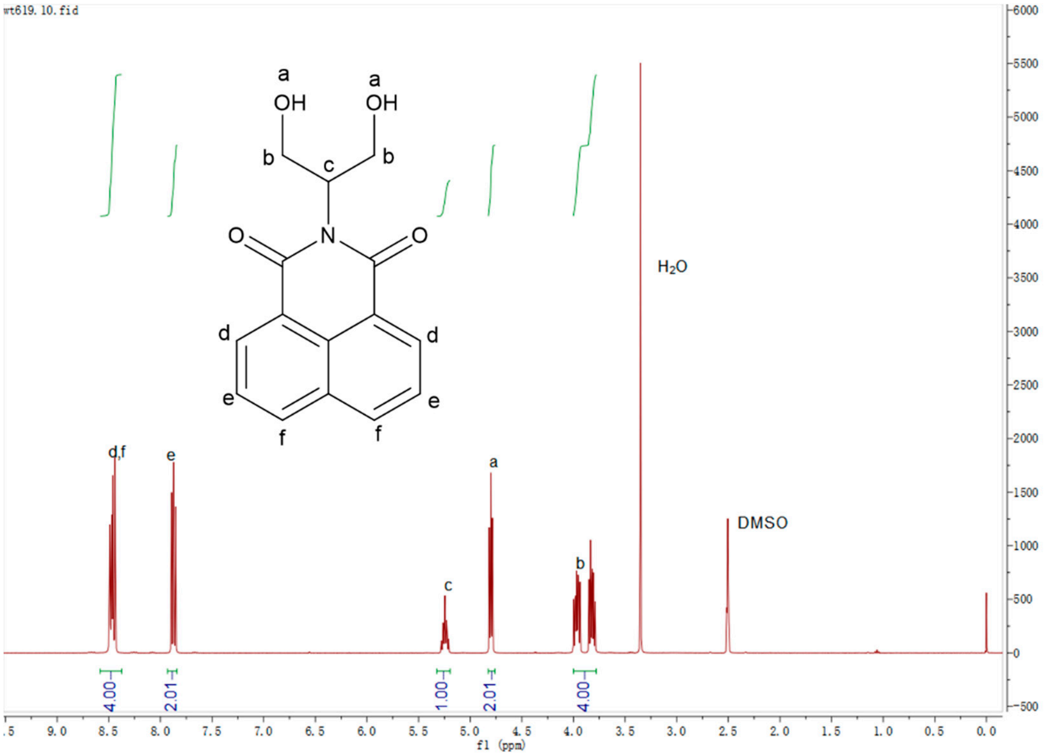

Figure S1. ^{1}H NMR spectrum of NI in DMSO

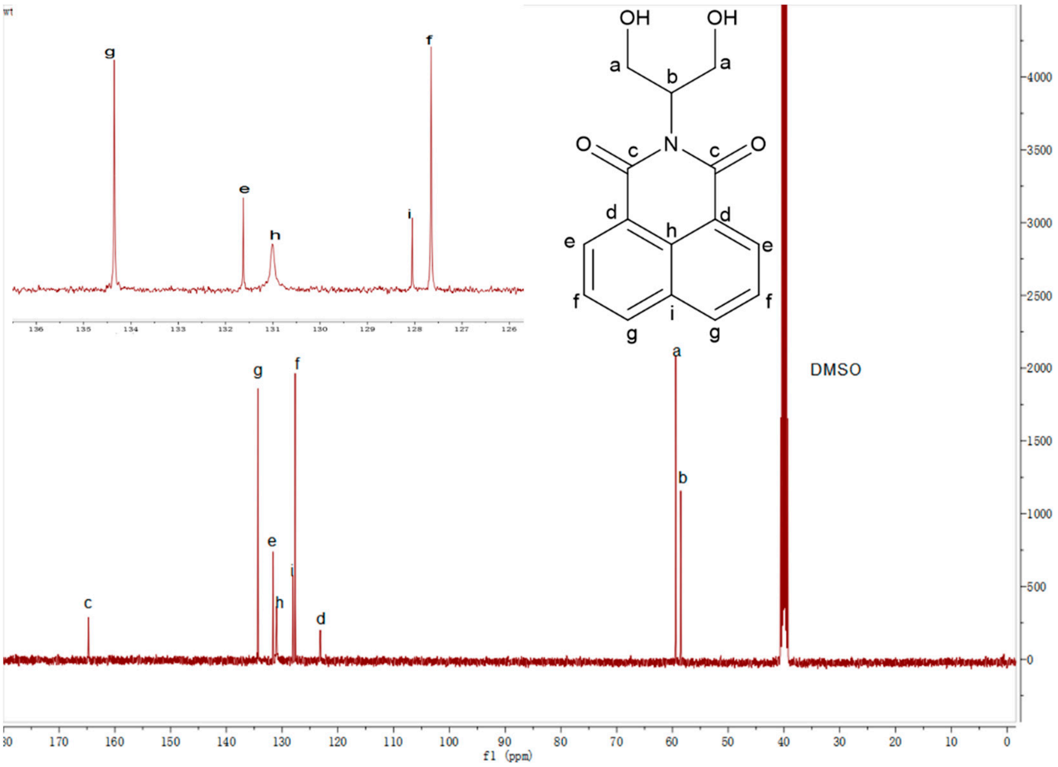

Figure S2. ^{13}C NMR spectrum of NI in DMSO.

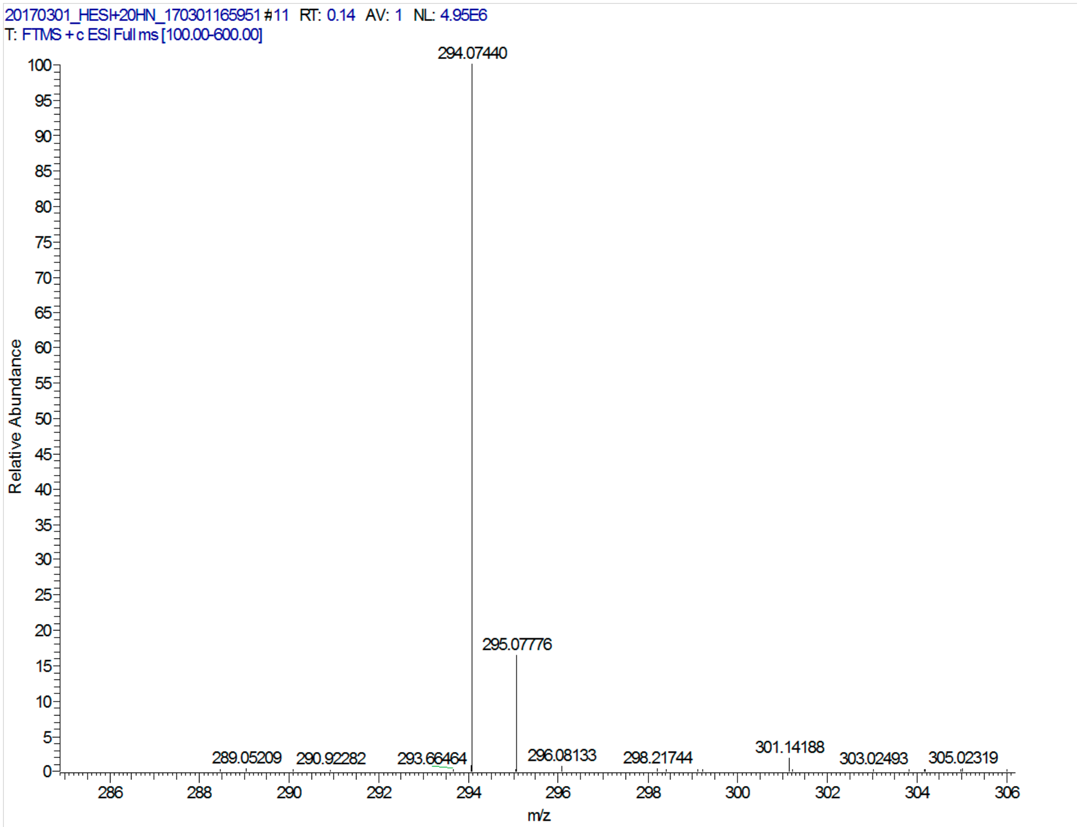

m/z	Intensity	Relative	Theo. Mass	Delta (mmu)	Composition
294.0744	4951230	100	294.07368	0.72	C15 H13 O4 N Na

Figure S3. MALDI-TOF-MS of NI.

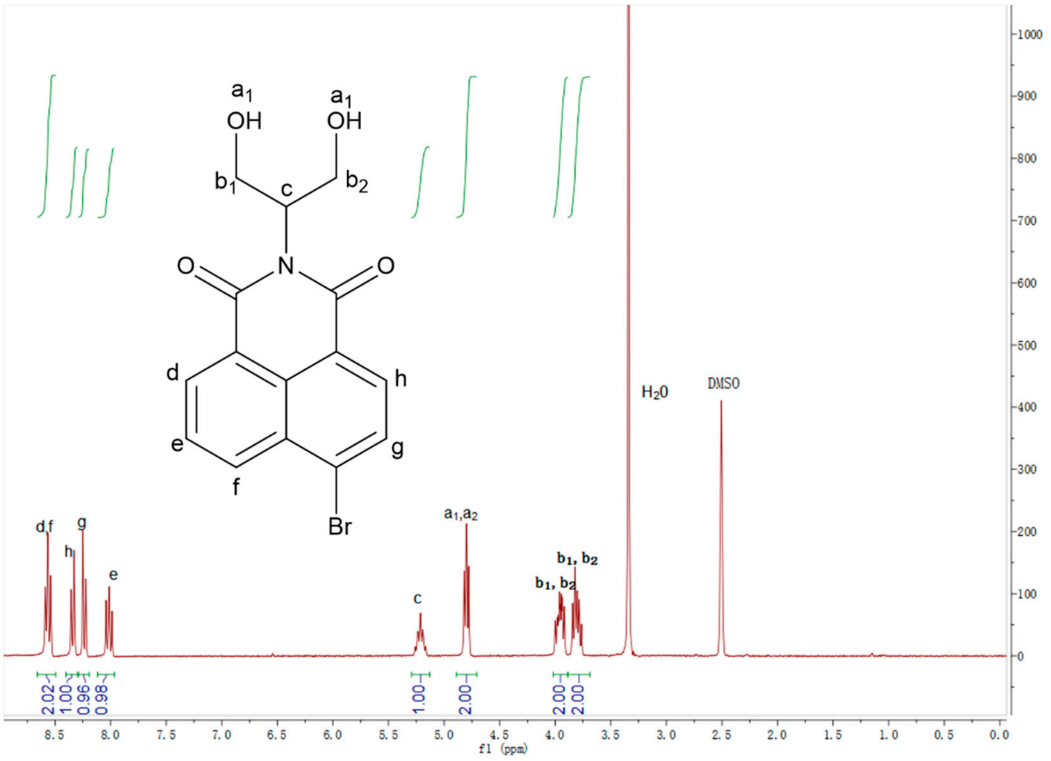

Figure S4. ^{1}H NMR spectrum of NIBr in DMSO.

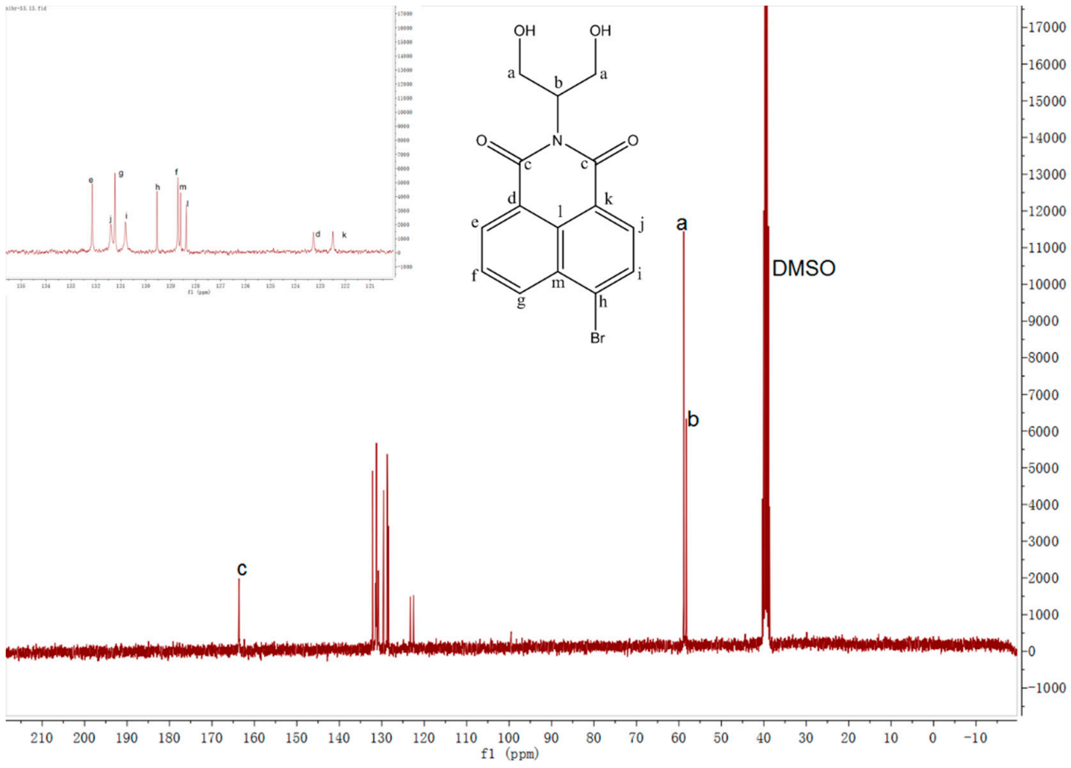

Figure S5. ^{13}C NMR spectrum of NIBr in DMSO.

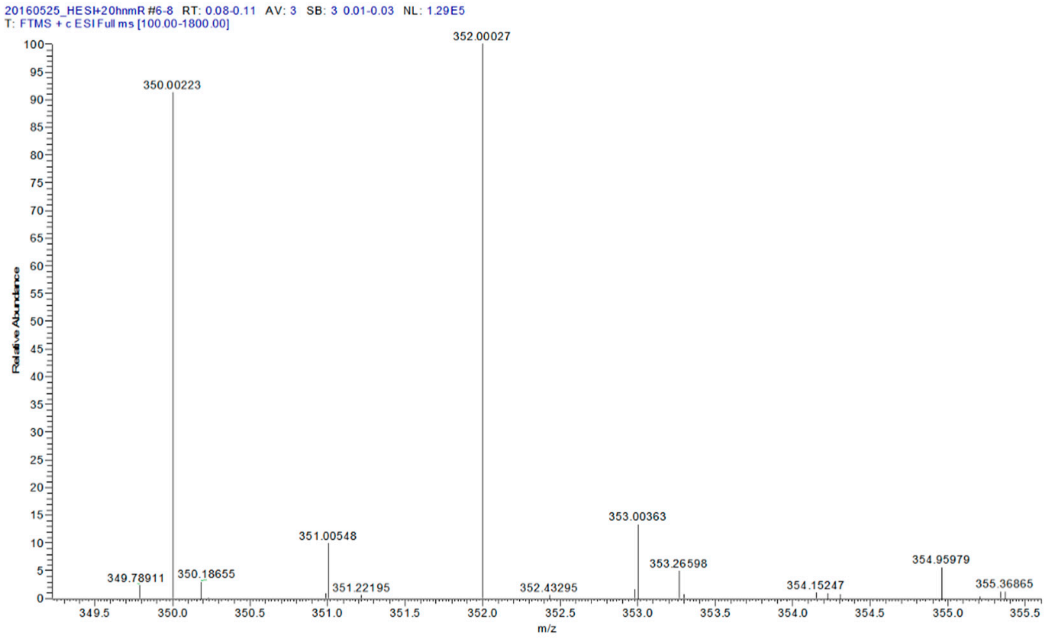

m/z	Intensity	Relative	Theo. Mass	Delta (mmu)	RDB equiv.	Composition
350.00223	117655.4	100	350.00225	-0.02	9.5	C15 H13 O4 N Br

Figure S6. MALDI-TOF-MS of NIBr.

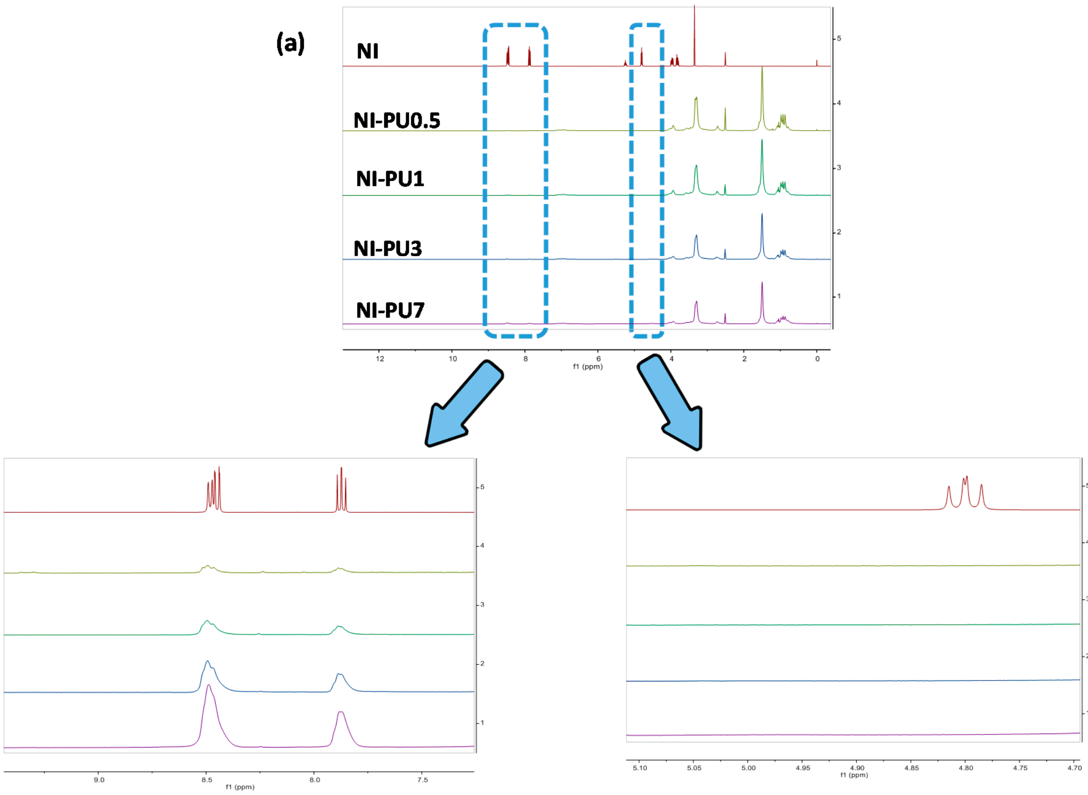

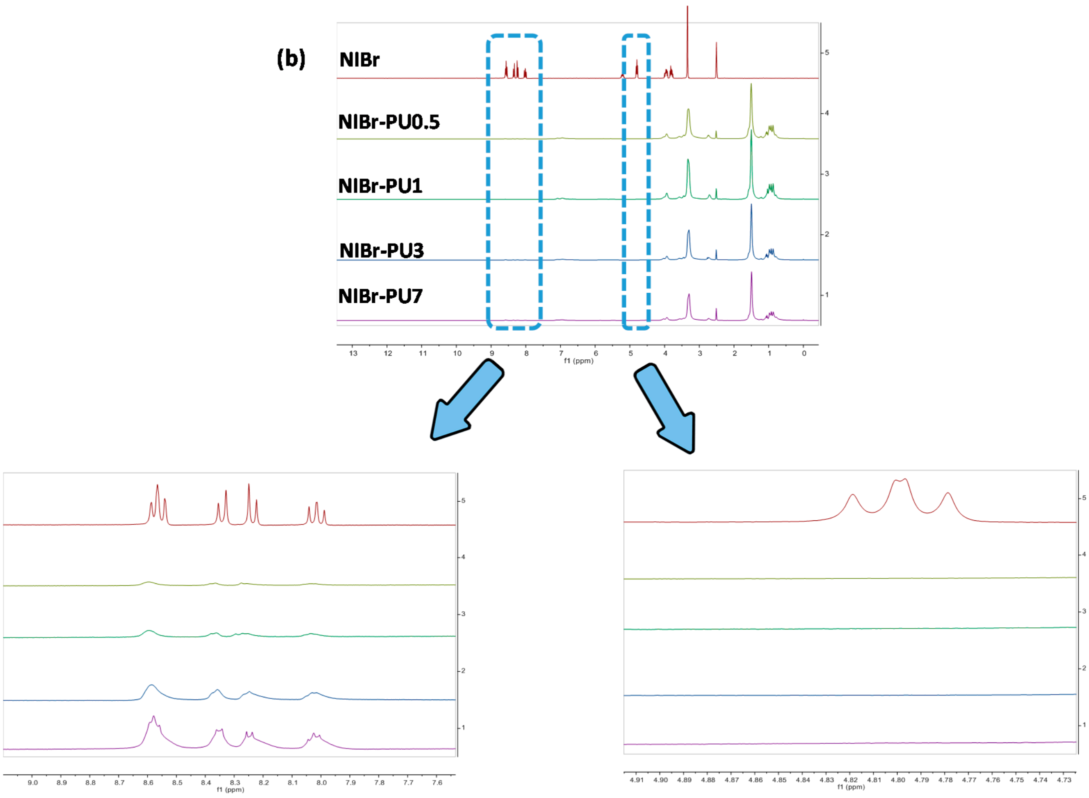

Figure S7. NMR analysis of NI-PUs (a) and NIBr-PUs in d_6-DMSO.

As the Figure S7(a) shows, the peaks around 8.47 and 7.87 ppm ascribed to the nuclear magnetic resonance of hydrogen atoms in phenyl rings appear in the NI-WPUs. With the increasing of dye loadings, the intensity of the peaks become more strongly, indicating the ratio of NI becomes higher. In addition, the peak belonging to hydroxyl is at 4.80 ppm. However, hydroxyl peak completely disappears in the NI-PUs, manifesting that hydroxyl has been incorporated into WPUs with a negligible loss, given that extremely high resolution of NMR. NMR characterization of NIBr-PUs is shown in Figure S7(b), the peaks centered at 8.66-8.49, 8.34, 8.24 and 8.01 ppm belongs to the nuclear magnetic resonance of hydrogen atom in phenyl rings. With the increase in the ratio of NIBr, there is the same trend of intensity of signal, which means more and more dyes are linked to the WPU covantly. Similiarly, the NMR peak at 4.80 belonging to hydroxyl dispears completely, indicating that NIBr is almost totally incorporately into the WPU.

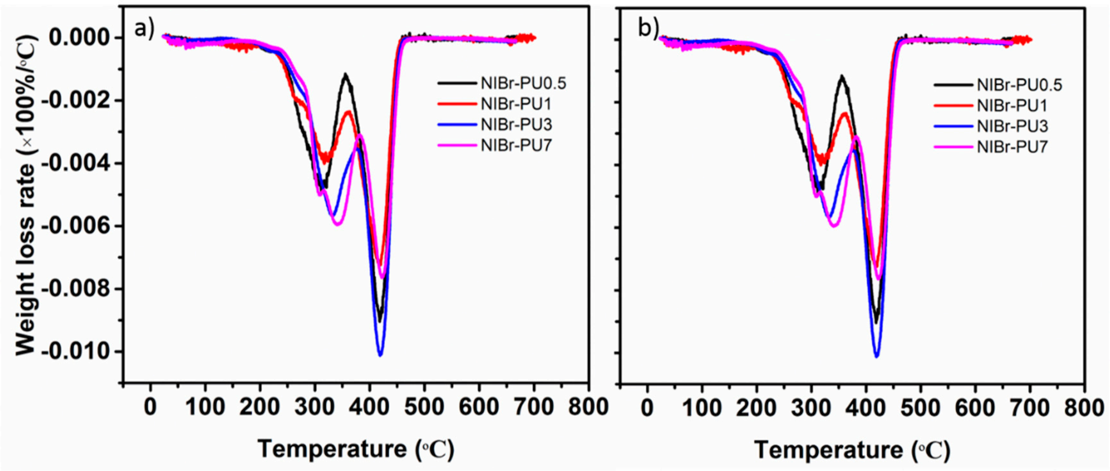

Figure S8. Derivative thermogravimetric (DTG) analysis of NI-PUs (a) and NIBr-PUs (b).

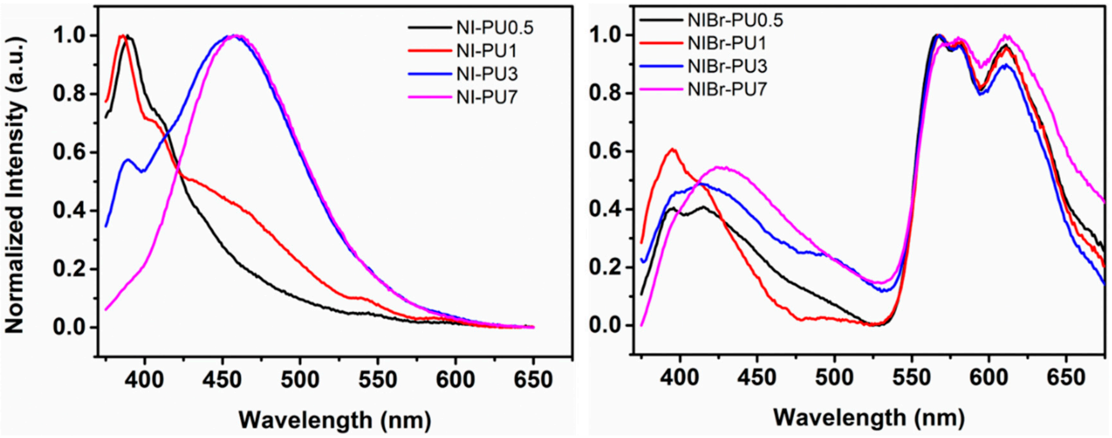

Figure S9. Steady-state emission spectra of different NI (a) and NIBr (b) concentrations in WPU under vacuum.

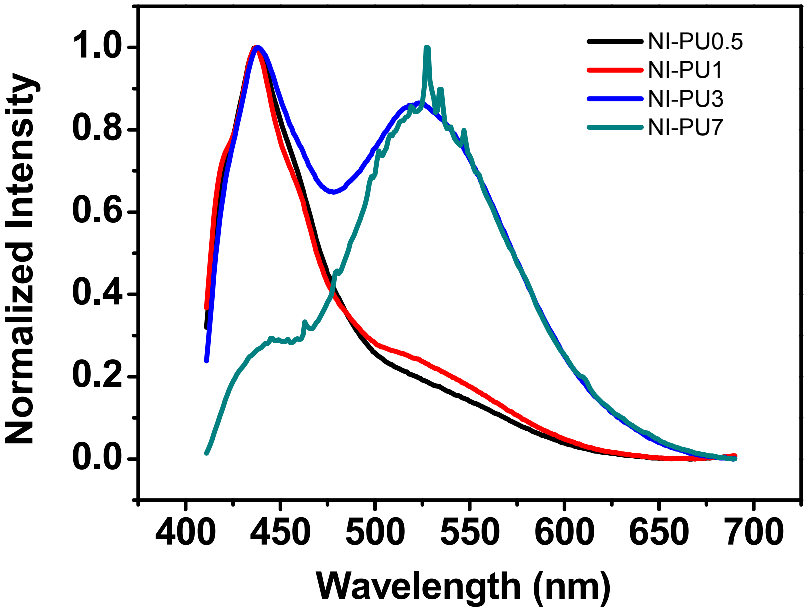

Figure S10. Normalized steady-state emission spectra of NI-PUs (λ_{ex} = 350 nm) in the emulsion state at room-temperature.

As the Figure S10 shows, with increased content of NI, the maximal emission peak shifts from 435 nm to 520 nm. Compared to NI-PU films, the spectra of NI-PU emulsions show a bathochromic shift to a greater degree, which may be ascribed to more entangled polymeric chain structure that makes singlet excitons interact with ground-state molecules more easily.

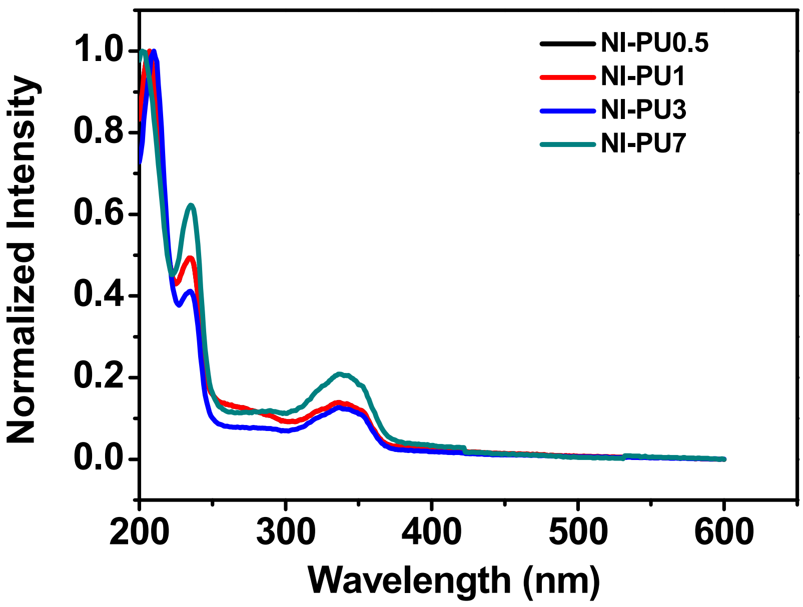

Figure S11. UV-Vis absorption spectra of NI-PUs in the emulsion state.

As the Figure S11 shows, all of the samples have the almost same absorption peak in the same condition, which means that NI-PUs have the same transition pathyway. Moreover, there are level-off tails in the visible spectral region, commonly obeserved in nanoparticle suspensions.

Table S1. lifetime data for NI-PUs in air and vacuum under room temperature

	λ^{a}_{air} (nm)	τ^{b}_{air} (ns)	λ^{c}_{vc} (nm)	τ^{d}_{vc} (ns)
NI-PU0.5	388	0.8	389	1.3
NI-PU1	386	0.8	386	1.2
NI-PU3	456	24.8	457	24.7
NI-PU7	462	26.1	459	25.7

a. steady-state emission maxima in air at room temperature (λ_{ex} = 365 nm). b. pre-exponent weight-averaged lifetimes in air at room temperature (nanoLED at λ_{ex} = 365 nm). c. steady-state emission maxima in vacuum at room temperature (λ_{ex} = 365 nm). d. pre-exponent weight-averaged lifetimes in vacuum at room temperature (nanoLED at λ_{ex} = 365 nm)

Table S2. Lifetime data for NIBr-PUs in air and vacuum under room temperature

	λ^{a}_{air} (nm)	τ^{b}_{air} (ns)	λ^{c}_{vc} (nm)	τ^{d}_{vc} (ns)	λ^{e}_{vc} (nm)	τ^{f}_{vc} (ms)
NIBr-PU0.5	393	0.1	396	0.1	566	5.40
NIBr-PU1	393	0.1	415	0.3	567	5.56
NIBr-PU3	425	2.9	420	2.0	569	4.95
NIBr-PU7	422	3.2	423	1.5	580	3.67

a. steady-state emission maxima in air at room temperature (λ_{ex} = 365 nm). b. pre-exponent weight-averaged lifetimes in air at room temperature (nanoLED at λ_{ex} = 365 nm). c. steady-state fluorescent emission maxima in vacuum at room temperature (λ_{ex} = 365 nm). d. pre-exponent weight-averaged lifetimes for fluorescence in vacuum at room temperature (nanoLED at λ_{ex} = 374 nm). e. steady-state phosphorescent emission maxima in vacuum at room temperature (λ_{ex} = 365 nm). f. pre-exponent weight-averaged lifetimes for phosphorescence in vacuum at room temperature (SpectraLED at λ_{ex} = 374 nm)

Table S3. Luminescent data for NI-PU emulsions in air at room temperature.

	λ[a] (nm)	τ[b] (ns)	Φ[c] (%)
NI-PU0.5	437	14.3	5.8
NI-PU1	438	16.4	6.2
NI-PU3	438	21.9	10.3
	520	23.3	
NI-PU7	523	24.4	10.7

a. steady-state emission maxima in air at room temperature (λ_{ex} = 365 nm). b. pre-exponent weight-averaged lifetimes in air at room temperature (nanoLED at λ_{ex} = 365 nm). c. relative fluorescent quantum yields under air at room temperature relative to that of quinine sulfate.

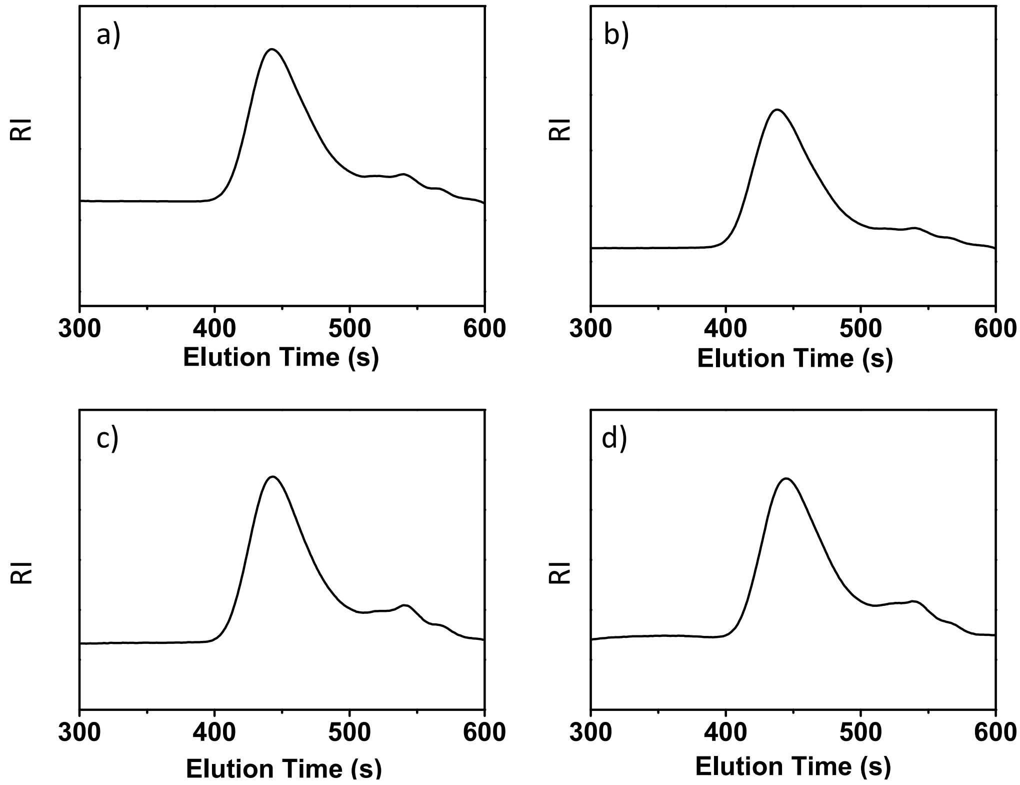

Figure S12. GPC traces for NI-PU0.5 (a), NI-PU1 (b), NI-PU3 (c), NI-PU7 (d) in THF.

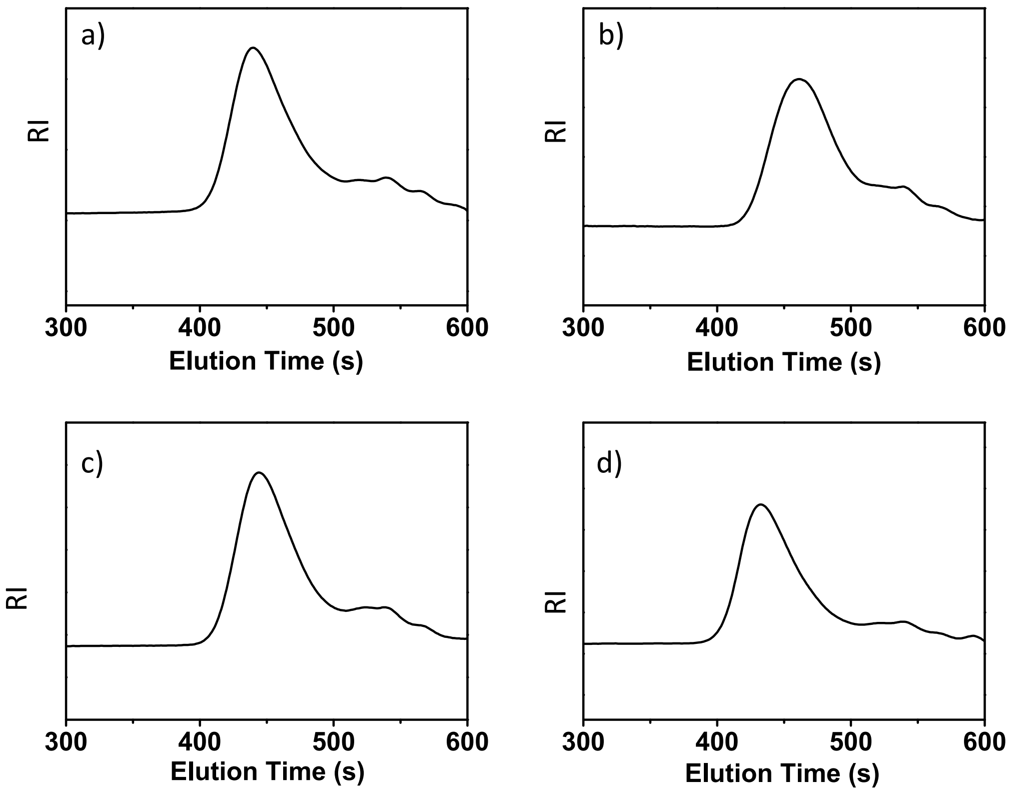

Figure S13. GPC traces for NIBr-PU0.5 (a), NIBr-PU1 (b), NIBr-PU3 (c), NIBr-PU7 (d) in THF with a flow rate of 0.3 mL/min^{-1}, calibrated with linear polystyrene standards.
